# New voices for a better society

**DOI:** 10.1073/pnas.2404579121

**Published:** 2024-04-24

**Authors:** Stephanie J. Diem, Baindu L. Bayon, Hussam Mahmoud, Mary L. Garcia-Cazarin, Michael J. Martin, Clare C. Rittschof, Patricia Silveyra, Alison Boland-Reeves, Dalal Najib, Flannery Wasson

**Affiliations:** ^a^New Voices, Cohort 2, National Academy of Sciences, Engineering, and Medicine, Washington, DC 20001; ^b^Nuclear Engineering and Engineering Physics Department, The University of Wisconsin-Madison, Madison, WI 53706; ^c^Department of Biology, Saint Mary’s College of California, Moraga, CA 94575; ^d^Department of Civil and Environmental Engineering, Colorado State University, Fort Collins, CO 80523; ^e^Department of Entomology, University of Kentucky, Lexington, KY 40546; ^f^Department of Environmental and Occupational Health, Indiana University, Bloomington, IN 46202; ^g^The National Academy of Sciences, Engineering, and Medicine, Washington, DC 20001

Recognizing that emerging leaders possess energy, insights, and communication skills needed to advance the dialog on our most pressing challenges, the US National Academies of Sciences, Engineering, and Medicine (NASEM) established the New Voices program in 2018 to bring diverse and innovative perspectives from early- and mid-career leaders to important dialogs around how science, engineering, and medicine are shaping the global future.

Over the past six years, 60 New Voices members have been selected to serve this mission by lending their time, energy, and expertise to enhance scientific and policy solutions to national and global challenges. Each interdisciplinary cohort serves a two-year term before transitioning to active alumni status and is supported by a staff Secretariat at NASEM and an Advisory Committee made up of senior experts dedicated to the New Voices mission.

The New Voices selection process is a merit-based, open competition from applications generated by interested individuals. New Voices members are selected through a competitive application review process, which includes submission of in-depth application materials, including three essays, two letters of reference, and examples of previous publications or other work. Applications are selected through two rounds of alumni reader scoring, finalist interviews, and selection panel deliberation to form a balanced, cohesive cohort.

In January 2024, New Voices admitted its third cohort of 21 new members. They join a larger network which includes 18 members of New Voices Cohort 1 (2018 to 2020) and 21 members of Cohort 2 (2021 to 2023). Over the next two years, New Voices members will meet to discuss and address key emerging challenges in science, engineering, and medicine, engage nationally with a wider group of young leaders from diverse groups, and attend international events to promote science diplomacy.

While each New Voices cohort defines its own focus, it also aims to build on the work of previous cohorts. During the term of service, the second New Voices cohort’s developed vision and mission statements centered around inclusive science and service. Its vision is that “...science is created by and belongs to all humanity. We envision a transformative scientific culture that fosters discovery, inclusion, and service.” Building on this vision, the mission is to “...accelerate the engagement of diverse and emerging leaders with the National Academies of Sciences, Engineering, and Medicine, the scientific community, and the public on scientific policy solutions to national and global challenges.” This statement is deliberately aspirational and reflects a belief that science must rise above an often troubled and exclusionary history. These statements guide cohort members’ approach to establishing evidence-based science and effectively communicating our perspectives on different issues.

Activities driven by New Voices members are naturally interdisciplinary due to the wide expertise, diverse backgrounds, and broad networks of cohort members. The New Voices program provides a platform to bring voices from diverse disciplines and sectors together to ensure science, engineering, and medicine are positioned to create a more informed, inclusive, and equitable society. While New Voices members bring diverse expertise and innovative perspectives to NASEM, they also define their areas of focus by building working groups, project committees, and initiatives that take an interdisciplinary approach to topics such as climate change, human rights, and academic reform. This group thus highlights the value of creating new venues for scientific engagement that extend beyond traditional structures and discipline boundaries. Moving forward, the NASEM, and the US scientific enterprise more generally will benefit from developing ways to sustain these types of transformative activities.

## An Unsiloed Interdisciplinary Approach Needed to Meet Complex Challenges and Grand Opportunities

The world we live in today is faced with many complex problems, including natural disasters, disease pandemics, climate change, and the associated social and economic costs of these events. From the 2018 Camp Fire in Paradise, CA, to the 2022 Hurricane Ian and Fiona over the Southeast, communities across the United States are facing extraordinary challenges as a result of climate change. Often knowledge of the impacts of these complex phenomena are siloed: Health care providers concerned about the health impact of heat waves rarely interact directly with power systems engineers concerned about the impact of heat waves on electricity transmission. However, building resilient systems in response to complex challenges such as climate adaptation requires combining knowledge of these impacts and an understanding of the interdependent nature of these systems (1). Members of New Voices Cohort 2 tackled this issue by producing a One Health webinar series focused on exploring linkages among environmental, human, and plant well-being. The webinar series focused on equitable and interdisciplinary approaches to advance technology and address climate change (2–4). To meet these challenges, New Voices members create work that brings together diverse early- and mid-career researchers in science, engineering, and medicine to accelerate the crossing of disciplinary boundaries. It also brings together broader perspectives to develop new technologies in an effective and equitable manner (5–7).

While discipline boundaries incentivized growth in the past, interdisciplinarity brings together subject matter experts to address complex scientific problems that characterize modern research goals (8). The interdisciplinary movement in part reflects the state of intellectual growth within disciplines, which are extending beyond foundational research questions and breaking new ground by adopting diverse technological advances (e.g., remote sensing and cheap and rapid nucleic acid sequencing, to name a few). The unacceptable reality has been a geographic and demographic exclusion of global resource-limited regions and underserved populations in advanced interdisciplinary research, which further broadens the disparity (9). Disciplines and funding agencies are also pushing researchers to revisit challenges that were historically neglected. These include incorporating gender, racial, and geographical diversity into health-related research studies as well as social and economic initiatives, for example, in efforts to develop and deploy modern energy systems. Advanced Research Projects Agency for Health (ARPA-H) is a new research funding agency that is focused on game-changing biomedical innovations that will reach all Americans. New federal funding priorities, such as the ARPA-H Novel Innovations for Tissue Regeneration in Osteoarthritis, are focused on addressing health disparities, as osteoarthritis is twice as common in women and has the highest prevalence in Black and Hispanic populations (10). The NSF Technology Innovation Partnerships Program focuses on meeting societal and economic needs by solving practical problems with a use-driven approach that is inclusive and that advances equity to provide tangible benefits for all people. NSF Regional Innovation Engines awards teams across the country to reach previously neglected regions that have not reaped the benefits of the most recent technology boom (11).

Recent philosophical and disciplinary shifts have given rise to one of the most broadly trained and interdisciplinary-minded generations of scientists to date (12). These scientists see value in reinventing the structure of scientific practice through interdisciplinary collaboration and are perhaps on the front wave of substantial change in research institutions (e.g., academia, national laboratories, and industry). In addition to an unusually broad context for their intellectual development, these scientists assumed their research positions at a time of increased social connectivity and a shift toward viewing scientific challenges (e.g., climate change and emerging disease) as countrywide and worldwide priorities. In the United States, this generation includes greater international presence and racial diversity than in years past (13). Beyond interdisciplinarity, new scientists realize that addressing complex problems will require a diverse and inclusive partnership and collective actions from the scientific community, government bodies, and the lay public (14). Perhaps as a result, many young scientists, even in the basic sciences, are adopting the service mantle that was historically more typical of applied disciplines (15). Funders are providing pathways for these scientists to more quickly build interdisciplinary multi-institution research teams. The New Voices program, by incorporating a motivated membership with immensely diverse disciplinary backgrounds, provides another type of venue for interdisciplinary action that does not fit within the traditional bounds of most scientific careers.

**Fig. 1.**
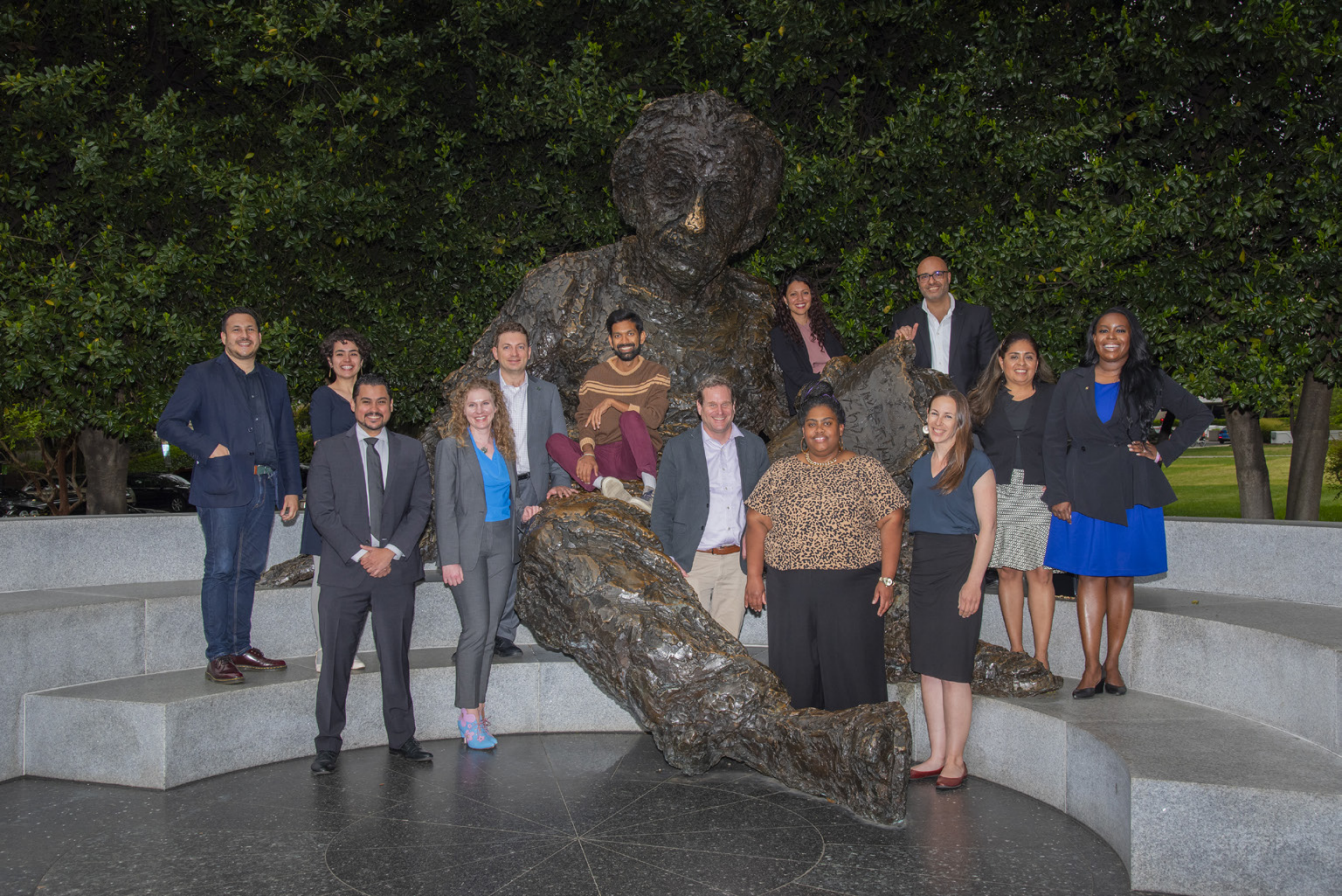
Cohort 2 members of the National Academies New Voices of Science, Engineering and Medicine. Pictured (*Left* to *Right*): Omar Asensio, Mahdieh Aghazadeh, Jesús Alvelo-Maurosa, Stephanie Diem, Umut Gurkan, Darshan Karwat, Michael Martin, Brandy Huderson, Patricia Silveyra, Elena Krieger, Hussam Mahmoud, Mary Garcia-Cazarin, and Baindu Bayon Paicely. Statue credit: Albert Einstein Memorial Statue; copyright 1978 by Robert Berks. Image credit: Risdon Photography.

Members of New Voices hold degrees ranging from health policy and pharmacology to physics and engineering. As an example, nearly one-third of the second cohort members are biomedical/healthcare professionals or engineers working on translational convergence research to enhance human health. Over half of the members have policy experience, either at their institutions or through a policy fellowship (e.g., AAAS, Mirzayan). Out of the 21 members of the second cohort (Fig. 1), half are first-generation immigrants. The diverse backgrounds of these members include South African, Egyptian, Turkish, Mexican, Indian, Sierra Leonean, Argentinean, American, and Iranian. New Voices members also represent institutions from most geographical regions of the United States and come from private industry, national laboratories, policy, government agencies, and diverse academic institutions including state land-grant universities, private universities, and historically black universities. Many New Voices members are not on the typical academic tenure track, and members completed their doctoral studies from 5 to 15 years before joining New Voices. The inclusion of many cultural perspectives is something that is acknowledged as necessary in science, engineering, and medicine when it was previously discouraged (16, 17). Young Academies around the world almost demand this diversity and inclusion to foster environments for new voices to truly be heard. Additionally, the global backgrounds, research, and networks of cohort members provide pathways to engage with broader networks to tackle global challenges facing humanity.

Cohort members bring historically excluded expertise and perspectives to the forefront of scientific dialog. In March 2019, the initial New Voices cohort hosted a public panel of leaders from government, industry, and academic sectors on diversity in science, engineering, and medicine at NASEM (17). Jane Zelikova is the cofounder of the groundbreaking organization 500 Women Scientists, focused on building an inclusive network of women scientists while addressing misogyny, xenophobia, and racism. Kelly Stevens cofounded the coalition behind the Fund Black Scientists movement (18), which focused on racial funding disparities in biomedical research. Enrico Castillo’s research aims to advance health equity and justice within biomedical research, having written about antiracist reforms to research policy and having codeveloped the Health Equity Research Impact Assessment (19–21). Umut Gurkan’s research is focused on basic and translational research, capacity-building, diverse workforce development, and community adoption of new medical technologies for the sustainable affordability of personalized medicine in underserved populations (22, 23). These initiatives are focused on problems of national importance in the United States specifically and benefit American science and society. This new era of Young Academies members is bold, empowered, and well equipped to lead this generation of scientists, engineers, and medical professionals.

The diverse backgrounds and experiences of New Voices members provide flexibility, adaptability, and the ability to be nimble, allowing cohorts to address complex challenges and provide evidence-based feedback to local and national leaders for improved responses to emerging national and global events. In March 2020, as the world was shutting down amid the global COVID-19 crisis, the first cohort of New Voices was at their annual meeting at Biosphere 2. In response to the public health emergency, the cohort shifted focus to the role the scientific community must take to provide an effective response (24) and designed the “Jennifer” chatbot as a proof-of-principle Artificial Intelligence system to learn whether public information from reputable sources can be more quickly and effectively organized and shared (25, 26).

Many of the New Voices’ initiatives have come from working groups formed within the cohorts. The Climate working group of Cohort 2 published an editorial highlighting the necessary skill set for engineers is expected to change as the climate changes. Specifically, engineers will need to 1) understand how climate and sustainability are linked to engineering design; 2) incorporate a wide range of disciplines into engineering solutions; 3) understand the ethics and justice dimensions of engineering; and 4) listen to and collaborate with diverse communities. Developing these skills requires immediate curriculum changes at all levels, from students to practicing engineers (27). Members of the Climate working group have integrated engineering and public health disciplines to examine the impact of increased intensity of heat waves on vulnerable populations, particularly as it relates to the decline in healthcare system capacity across the United States. The results of the analysis were used to devise recommendations, soon to be published, toward lowering the expected impact and improving public health outcomes throughout the country. Additionally, Hussam Mahmoud, in partnership with the Inter Academy Partnership (IAP), the Global Young Academy, and the Institute for European Environmental Policy convened a side event at COP 28 to discuss how senior and Young Academies around the world can work together to address climate challenges. During the panel, Mahmoud highlighted the various ongoing climate-related initiatives by the New Voices, including the analysis of healthcare resilience as a central pillar for addressing heat waves.

“Disrupting Academia,” one of the working groups to come from New Voices Cohort 2, is reexamining what existing structures in academia are working for scientists and engineers, and which will need rethinking. This group’s long-term goals are to generate guidelines to support scientists and engineers who reflect the population of the United States, to create and present data to support guidelines, to endorse careers that are not centered around academia, and finally, to amplify the stories of those who are underrepresented in science, engineering, and medicine. This group wishes to consider moving away from traditional metrics for success, supporting marginalized and underrepresented faculty through the implementation of activities and development of guidelines that consider their unique needs, generating new policies, and disseminating relevant resources to academic communities. Most recently, the Disrupting Academia working group collaborated with the NASEM Government-University-Industry Research Roundtable to host *Bridging the Gap: Overcoming the Hurdles in Innovation Culture Across Sectors* (28). In this session, expert panelists discussed their experiences navigating commercialization and the systems that enable or hinder progress in the science and tech entrepreneurship spaces. Ultimately, the products of this working group will increase engagement at institutions and encourage them to examine their policies/procedures/practices that may perpetuate the inequitable impact of science, engineering, and medicine innovations.

A third working group, “Science, Trust, and Human Rights” is concerned with issues in how science interacts with society. The group arose out of the concerns of New Voices members about issues including the growing urgency of bridging communication gaps between the public and scientific community around COVID; the challenges faced by scientists displaced by war and political persecution; and the need to expand pathways for engineers and scientist to contribute to building a better world. The working group is partnering with the NASEM Committee on Human Rights. Working together with the New Voices cohort as a whole, the working group identified other closely related issues, including equity and inclusion in US science, building equitable international collaborations, and defining human rights impacts of climate and pathways toward a just energy transition, as critical areas for the scientific community. A representative of this working group, Michael Martin, presented the concerns of New Voices members in the opening Young Scholars session of the International Human Rights Network of Academies of Scholarly Societies biannual meeting in Pretoria, South Africa. Members of this group are continuing to work directly with the Committee on Human Rights to identify how scientists, engineers, and medical practitioners can integrate an understanding of human rights impacts into their work.

## Long-Lasting and Global Impacts on New Voices

There is a growing movement of Young Academies around the world with more than 54 National Young Academies globally (29–31). Like New Voices, many Young Academies are affiliated with established National Academies. New Voices members are given opportunities to bring the voices of early- and mid-career scientists to national and global discussions for the advancement of science diplomacy, advance the dialog between science and society, and engage in interdisciplinary work with each other as well as members of the global Young Academy community. For most of this cohort, and the other cohorts, this is oftentimes the first opportunity - we are not typically funded or rewarded to engage in interdisciplinary research in our individual networks globally.

**Fig. 2.**
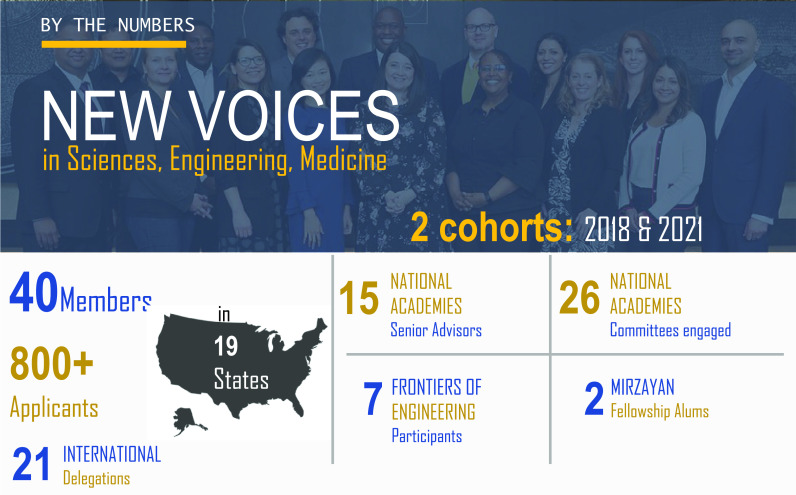
Highlights of the New Voices program as of December 2022. Image credit: Eikon Photography.

New Voices members bring their diverse expertise and perspectives through formal roles in the NASEM and are engaged in leading their own initiatives and events (Fig. 2). Since 2020, New Voices members have served on more than 21 NASEM committees and contributed to over 17 convening activities. NASEM has sent delegations of New Voices members to over 27 major international and domestic events to represent young US scientists on the global stage, including the Science and Technology in Society Forum in Kyoto, Japan, the World Laureates Association Forum in China, COP28 in Dubai, and the World Science Forum, an international conference series on global science policy, in Budapest, Hungary. New Voices members from Cohort 1 envisioned and designed a geographic information system (GIS)-enabled interactive database for maintaining and growing our network of vetted emerging leaders. Working with a team from Mapbox, the prototype (32) GIS database displays secondary connections among network members in addition to their primary affiliations, social media presence, and other categories. The network tool is currently being expanded by Cohort 2 to include a broader network of early-career peers. New Voices members also can nominate other emerging experts in their networks to be considered for NASEM committees. This tool is intended to support NASEM in the future selection of experts from diverse backgrounds to be engaged in different activities. Members of Cohort 1 provided new perspectives on science communication centered on personal storytelling (33). This resulted in the publication of two books by cohort 1 member Faisal Hossain, titled “The Secret Lives of Scientists, Engineers, and Doctors” (34, 35). In these books, members of Cohort 1 along with scientists in their professional networks were depicted as cartoons next to personal stories aimed to inspire readers of all ages.

The engagement of Cohort 1 members in various NASEM activities has undoubtedly contributed to the advancement of their careers. Alumni of the previous cohorts have been recognized as today’s leaders: for example, Asmeret Berhe has been appointed as the Director of Office Science at the Department of Energy (36) and was elected to the National Academy of Engineering in 2023. New Voices alumna, Frances Colón, has also been appointed to the President's Council of Advisors on Science and Technology (37). Ali Nouri has been appointed as the Deputy Assistant to the President and Deputy Director of the Office of Legislative Affairs at the White House. Others have achieved important leadership positions in their institutions, such as Associate Vice Provost (Joel Baumgart at Emory University), Department Chair (Patricia Silveyra at Indiana University Bloomington), and Director of a Medical Science Training Program (Olujimi Ajijola at UCLA). Many have received awards and recognition for their contributions to projects piloted by New Voices.

**Fig. 3.**
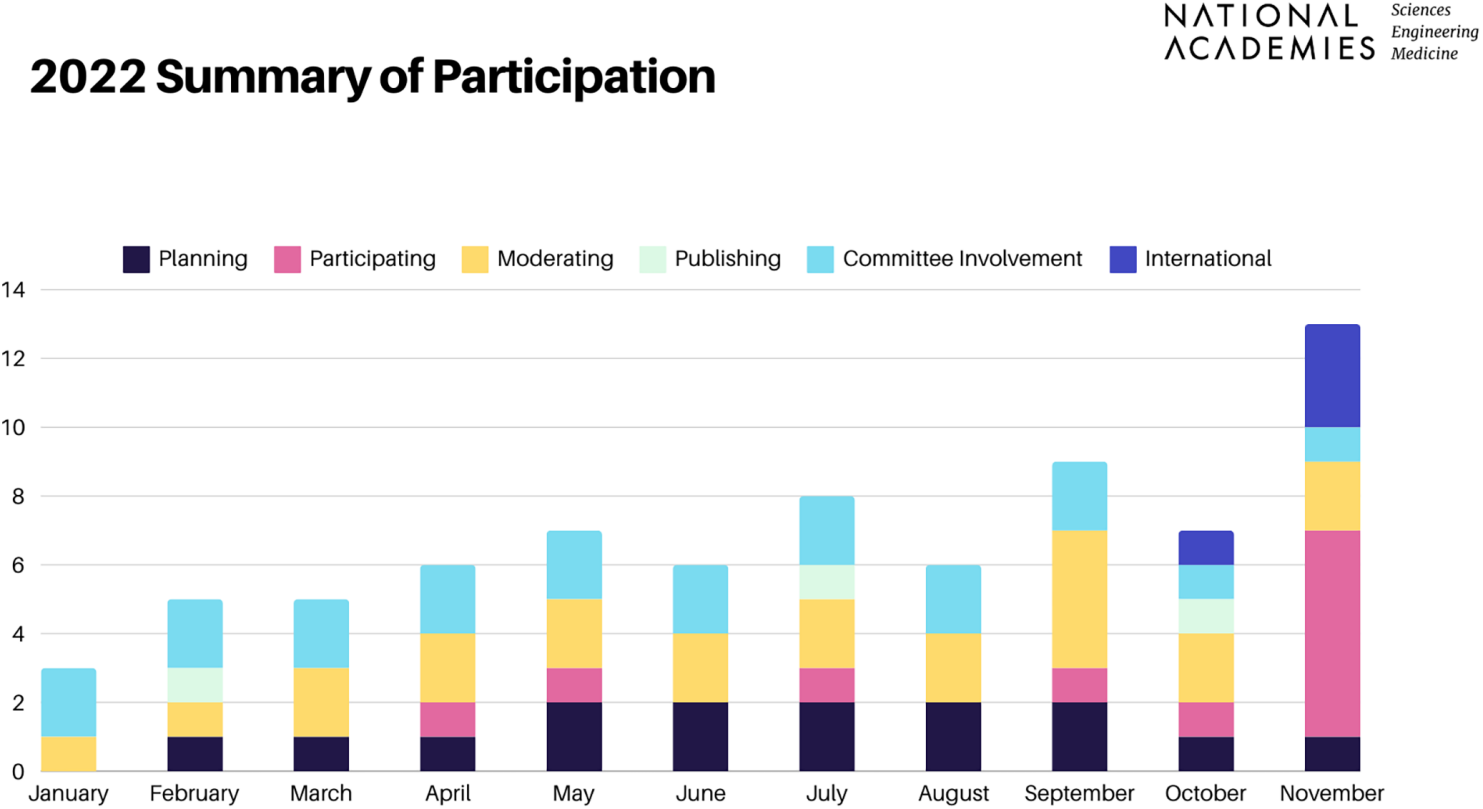
2022 Summary of participation for New Voices members in National Academies activities. Data compiled by Flannery Wasson, Tracy Sahay, Jesús Alvelo-Maurosa, Umut Gurkan, Hussam Mahmoud, and Kelly Stevens.

Leveraging the momentum of Cohort 1 and with the support of NASEM program officers and staff, there has been an increased uptick in the level of engagement of Cohort 2 members in current NASEM projects (Fig. 3). According to a December 2022 survey of both cohorts, while 83% of the surveyed members had not engaged with NASEM before their appointment, 80% of them are now actively involved. New Voices members have contributed to the scientific enterprise of NASEM through organizing and participating in different activities. For example, Elena Krieger is a member of the committee on Hazard Mitigation and Resilience Applied Research Topics (38), Hussam Mahmoud chaired a NASEM workshop on Benefits, Applications and Opportunities of Natural Infrastructure (39), Mahdiah Aghazadeh was a committee member for the Endless Frontier Symposium 2022: Research and Higher Education Institutions for the Next 75 Years (40) where Darshan Karwat was a featured speaker, and Mary Garcia-Cazarin moderated a panel for the NASEM Breaking Glass: Advancing Women Leaders in Science event (41).

New Voices members/alumni have also promoted the NASEM’s science diplomacy efforts to engage with the Arab and Muslim world through organizing and participating in the Arab-American Frontiers program, and building scientific capacity in Africa by organizing and participating in the first and second US-Africa Frontiers symposia, and promote diversity in STEM through service in the committees of the Board on Higher Education and Workforce, the Roundtable on Black Men and Women in STEM, and participate in Government University Industry Research Roundtable events. Additionally, New Voices members from Cohort 2 were able to connect with other Young Academies by serving on the organizing committee of the Triennial Conference of the IAP and Worldwide Meeting of the Young Academies, which took place at Biosphere 2 in Arizona in November 2022 (42). This is the first time the IAP and the Young Academies held their conferences jointly. Stephanie Diem, Patricia Silveyra, and Hussam Mahmoud played different roles as conference and session organizers and presented the major milestones and accomplishments achieved by New Voices members and cohorts so far. Umut Gurkan was the US cochair of the 2023 Arab-American Frontiers in Science, Engineering and Medicine Symposium and served on the NASEM committee for the “On Leading a Lab: Strengthening Scientific Leadership in Responsible Research” workshop. To further support the growing global movement of young academies, New Voices will be hosting the Global Young Academy meeting in 2024 at the National Academies in Washington, DC. The large increase in National Academies engagement, science diplomacy, and global activities can broaden career paths and/or research of New Voices members.

## Paving the Path Forward for Future New Voices

As with many unique initiatives, there are opportunities for the program to improve. The momentum of New Voices initiatives can be achieved by continuing to form collaborations with new units within the NASEM (e.g., the National Academy of Medicine, the Climate Crossroads Initiative, etc.). Additionally, the impact of each cohort’s efforts can be strengthened through overlapping cohort members and engaging alumni and a wider network of other emerging STEM leaders in the United States (43). As with many service organizations, communicating expectations and opportunities for New Voices members in various NASEM activities can help minimize the orientation time. Recently, the National Academies have launched a website and videos (44) explaining the volunteer process which helps reduce the time spent by new New Voices members and NASEM volunteers learning about the details of serving on committees and understanding the level of commitment needed. It is essential that those interested in serving in programs like New Voices understand the level of commitment needed to contribute to the variety of service activities members are involved in—from New Voices initiatives to serving on National Academies committees.

Looking ahead, the New Voices program will provide a unique opportunity for a diverse cohort of early- and mid-career scientists, engineers, and doctors to address emerging scientific challenges with an interdisciplinary approach in service to NASEM, society, and the broader scientific community. This program provides an invaluable experience for New Voices members to broaden their networks to engage with different scientific fields and science policy. Programs like New Voices are progressive and generate unique solutions that will require funding. Financial resources are key to supporting high-performing cohorts and transforming this pilot initiative into a multiyear program with long-term impact. This program is a wonderful example of the impact that is possible when creative and diverse young researchers are provided a venue to enact their values and perspectives.
